# Correction: Single-cell analysis of the cellular heterogeneity and interactions in the injured mouse spinal cord

**DOI:** 10.1084/jem.2021004007232025c

**Published:** 2025-08-11

**Authors:** Lindsay M. Milich, James S. Choi, Christine Ryan, Susana R. Cerqueira, Sofia Benavides, Stephanie L. Yahn, Pantelis Tsoulfas, Jae K. Lee

Vol. 218, No. 8 | https://doi.org/10.1084/jem.20210040 | June 16, 2021

In the original legend of Fig. 5, the descriptions for panels A, B, and C were mislabeled, and the related citations in the text used the wrong panel letters. In the original article, panel A described panel C, panel B described panel A, and panel C described panel B. In addition, the Fig. 5 B key was erroneously labeled. The orange and blue bars have been corrected to read "APOE^lo^" and "APOE^hi^," respectively. The original and corrected Fig. 5 and its legend are shown here, as are the corrected citations in the Results section and the Fig. 3 legend, which are shown in bold and underlined. The conclusions of the article remain unchanged.

The Fig. 5 legend and citation errors appear in print and in PDFs downloaded before July 31, 2025. The Fig. 5 B key errors appear in print and in PDFs downloaded before August 6, 2025.

## Results

**Myeloid analysis reveals temporal changes in macrophage and microglial subtypes** (*fifth paragraph*)

To validate the presence of chemotaxis-inducing and inflammatory macrophage subtypes in vivo, we performed immunohistochemistry using antibodies against CD63 and APOE to spatially validate our results (**Fig. 5 C**). While CD11b^+^ myeloid cells were present at all time points, CD11b^+^/CD63^+^ cells started to appear by 3 dpi. By 7 dpi, many CD11b^+^/CD63^+^ cells that were either APOE^lo^ (chemotaxis-inducing) or APOE^hi^ (inflammatory) were present at the lesion core. Since graded expression based on fluorescence intensity is difficult to quantify using immunohistochemistry, we used flow cytometry to quantify the relative proportion of these two macrophage subtypes. After separating monocytes/macrophages from other leukocytes including microglia (see Materials and methods), we isolated macrophages based on CD63^hi^ expression (**Fig. 5 A**). Further separation on APOE and CD11b expression revealed two distinct clusters that were consistent with chemotaxis-inducing (CD63^hi^/CD11b^med^/APOE^lo^) and inflammatory (CD63^hi^/CD11b^hi^/APOE^hi^) subtypes (**Fig. 5 A**). Similar to our sequencing data, the CD63^−^ myeloid cells were the predominant population at 1 dpi, and decreased by 7 dpi, whereas inflammatory macrophages (APOE^hi^) were the most represented macrophage subtype at 7 dpi (**Fig. 5 B**). Therefore, both immunohistochemistry and flow cytometry data support the molecular identification of chemotaxis-inducing and inflammatory subtypes and their temporal progression after SCI in vivo. In summary, analysis of myeloid cells reveals novel subtypes of microglia and macrophages during SCI progression.

**Figure 3:**
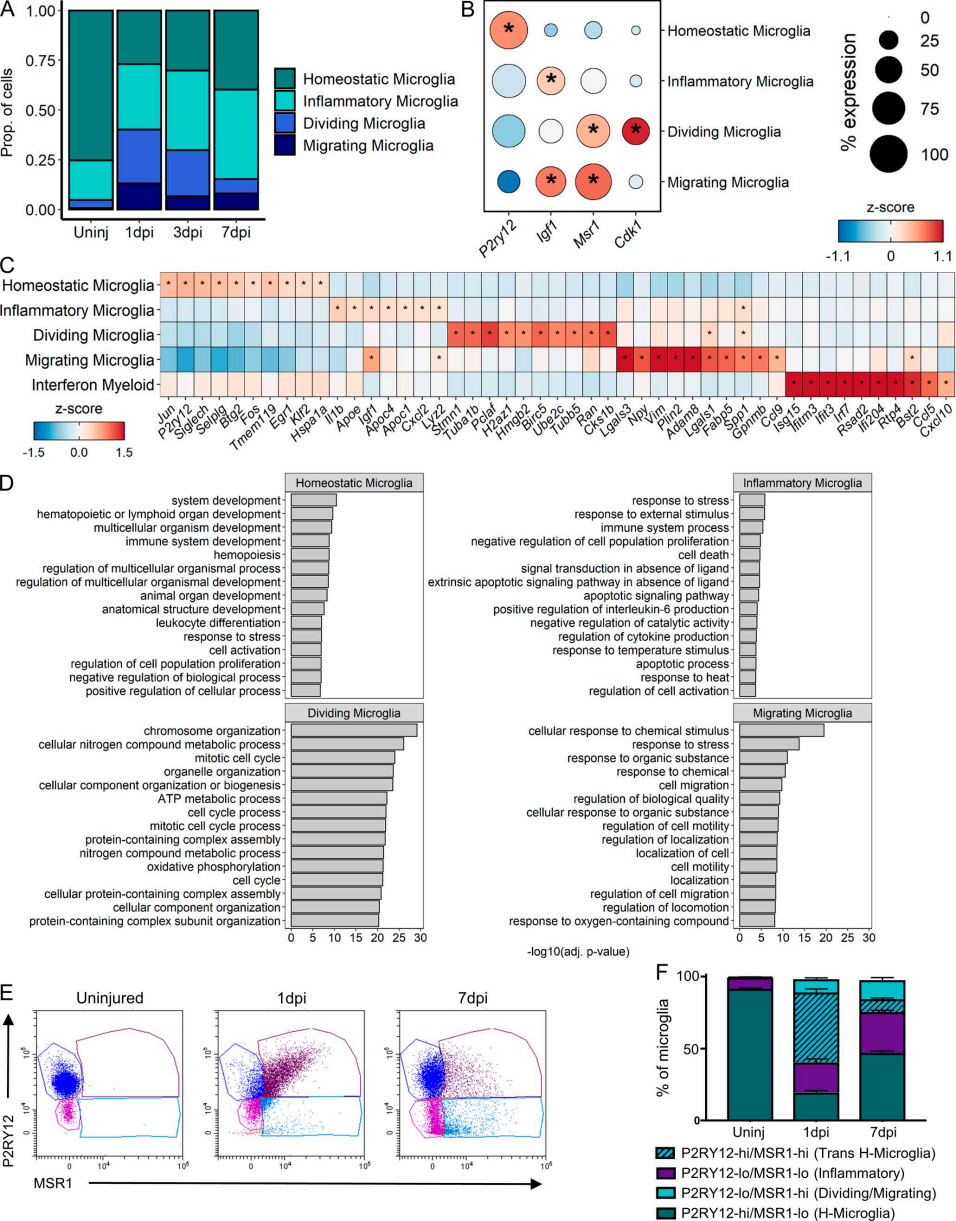
**Molecular and temporal profile of microglia subtype heterogeneity acutely after SCI. (A)** Proportion of each microglia subtype among all microglia at each time point. **(B)** Dot plot of marker genes that differentiate nonhomeostatic microglia subtypes from homeostatic microglia. Color of dots represents z-scored expression level, and size of dots represents percentage of cells with at least one UMI detected per gene. **(C)** Heatmap of the highest DEGs per microglia subtype. Color represents z-score expression level. Inset stars in B and C indicate statistically significant greater expression compared with all other microglia combined (adjusted P value < 10^−10^; Wilcoxon rank-sum with Bonferroni correction; see Materials and methods). **(D)** GO enrichment analysis of the DEGs per subtype. GO biological process terms displayed along y axis, and –log_10_ (Bonferroni-corrected P values) displayed along x axis. **(E)** Scatter plot of flow cytometry analysis of microglia in the uninjured spinal cord and injured spinal cord at 1 and 7 dpi. Y axis and x axis display fluorescence intensities of P2RY12 and MSR1, respectively. After removing nonviable cells and gating out Ly6G^+^ cells, CD45^med^/CD11b^+^ microglia were selected from other leukocytes for further gating along P2RY12 and MSR1 quadrants (see Materials and methods and **Fig. 5 A**). **(F)** Quantification of proportion of microglia subtypes in flow cytometry as gated in E. adj., adjusted; H-microglia, homeostatic microglia; Prop., proportion; Uninj, uninjured; Trans, transition; Lo, low; Hi, high.

**Figure 5:**
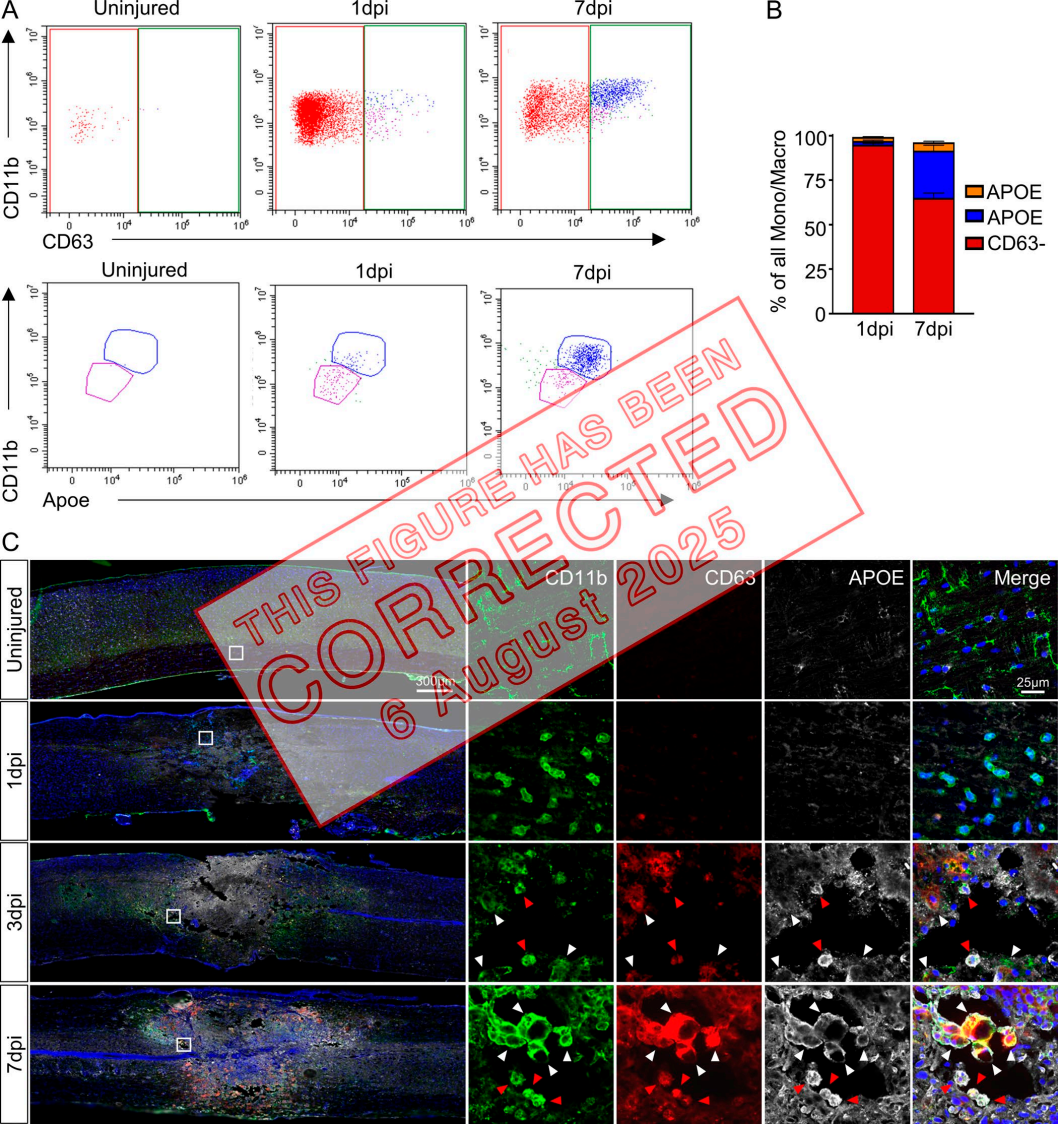
**In vivo validation of chemotaxis-inducing and inflammatory macrophages after SCI. (A)** Immunohistochemical validation of chemotaxis inducing and inflammatory macrophages and their temporal changes after SCI. Injured spinal cord tissue shows a steady increase in macrophages (CD11b^+^/CD63^+^) over time. APOE expression differentiates between chemotaxis-inducing (CD11b^+^/CD63^+^/APOE^lo^) and inflammatory (CD11b^+^/CD63^+^/APOE^hi^) macrophages. Chemotaxis-inducing macrophages are identified by white arrowheads, while inflammatory macrophages are identified by red arrowheads. Boxed regions on the left are shown in higher magnification on the right. Representative images from a total of three biological replicates per each time point. Scale bars in merged images are 300 µm, and scale bars in magnified images are 25 µm. Blue is DAPI to label nuclei. **(B)** Scatter plot of flow cytometry analysis and gating strategy (see Materials and methods for description) to quantify relative proportions of the two macrophage subtypes. After excluding neutrophils and lymphocytes, monocytes/macrophages were separated from microglia based on CD11b and CD45. After gating for CD63^hi^ macrophages, separation based on CD11b and APOE resulted in CD11b^med^/APOE^lo^ (chemotaxis-inducing macrophages) and CD11b^hi^/APOE^hi^ (inflammatory macrophages) clusters. **(C)** Quantification showed increased proportion of CD11b^hi^/APOE^hi^ macrophages at 7 dpi compared with 1 dpi. *n* = 5 biological replicates at each time point for flow cytometry experiment. Macro, macrophage; Mono, monocyte.

**Figure 5:**
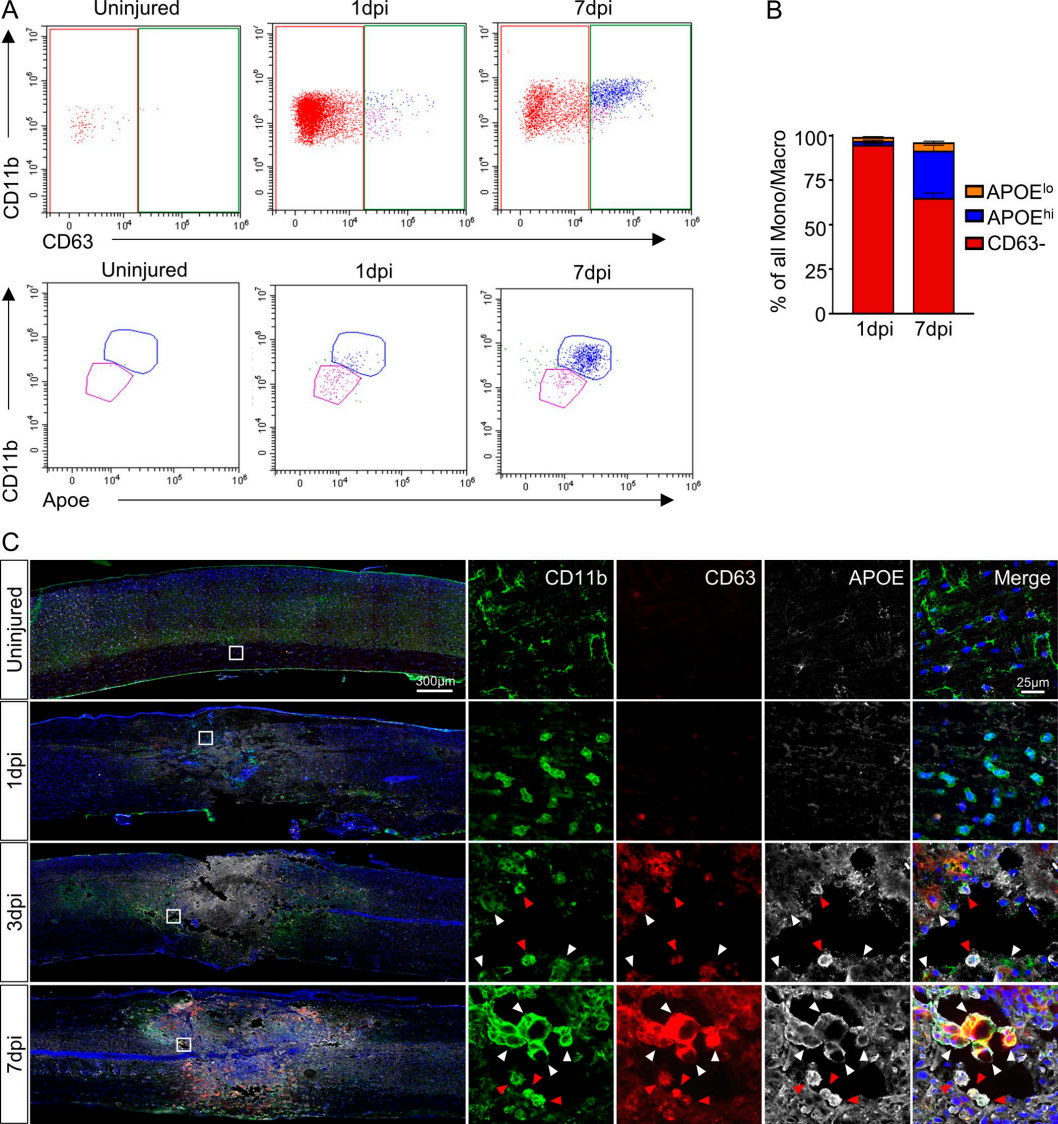
**In vivo validation of chemotaxis-inducing and inflammatory macrophages after SCI. (A)** Scatter plot of flow cytometry analysis and gating strategy (see Materials and methods for description) to quantify relative proportions of the two macrophage subtypes. After excluding neutrophils and lymphocytes, monocytes/macrophages were separated from microglia based on CD11b and CD45. After gating for CD63^hi^ macrophages, separation based on CD11b and APOE resulted in CD11b^med^/APOE^lo^ (chemotaxis-inducing macrophages) and CD11b^hi^/APOE^hi^ (inflammatory macrophages) clusters. **(B)** Quantification showed increased proportion of CD11b^hi^/APOE^hi^ macrophages at 7 dpi compared with 1 dpi. *n* = 5 biological replicates at each time point for flow cytometry experiment. Macro, macrophage; Mono, monocyte. **(C)** Immunohistochemical validation of chemotaxis-inducing and inflammatory macrophages and their temporal changes after SCI. Injured spinal cord tissue shows a steady increase in macrophages (CD11b^+^/CD63^+^) over time. APOE expression differentiates between chemotaxis-inducing (CD11b^+^/CD63^+^/APOE^lo^) and inflammatory (CD11b^+^/CD63^+^/APOE^hi^) macrophages. Chemotaxis-inducing macrophages are identified by white arrowheads, while inflammatory macrophages are identified by red arrowheads. Boxed regions on the left are shown in higher magnification on the right. Representative images from a total of three biological replicates per each time point. Scale bars in merged images are 300 µm, and scale bars in magnified images are 25 µm. Blue is DAPI to label nuclei.

